# Effect of Humidity on the Mobilities of Small Ions in Ion Mobility Spectrometry

**DOI:** 10.1021/acs.analchem.3c00435

**Published:** 2023-05-23

**Authors:** Izabela Wolańska, Krzysztof Piwowarski, Edyta Budzyńska, Jarosław Puton

**Affiliations:** Faculty of Advanced Technologies and Chemistry, Military University of Technology, ul. gen. Sylwestra Kaliskiego 2, 00-908 Warsaw, Poland

## Abstract

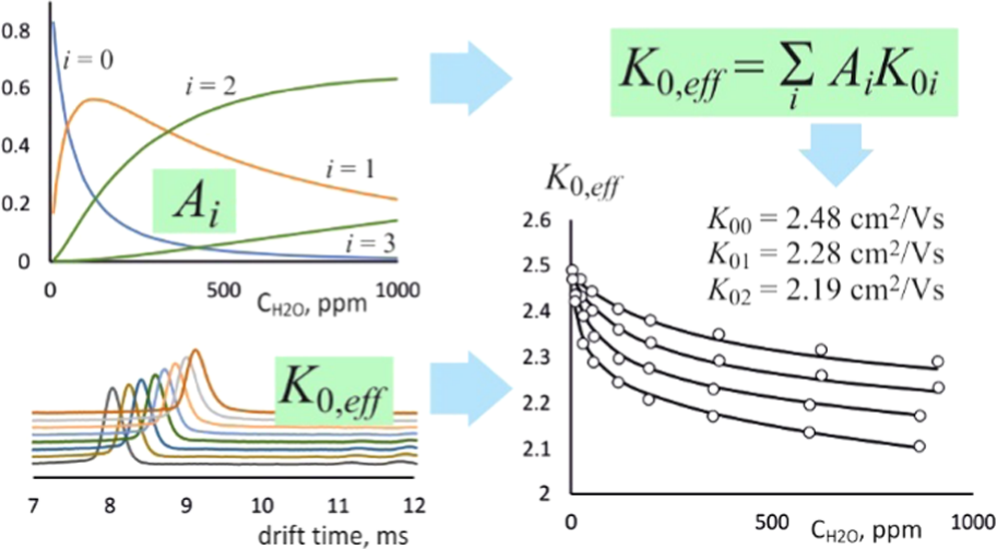

Ions in the ion mobility spectrometry (IMS) are mostly hydrated. A single peak in the drift time spectrum is usually generated by a mixture of ions differing in the number of attached water molecules. Under real IMS detector operating conditions, ions change their composition during movement in the drift region due to the changes in the number of water molecules attached to the ion. The impact of water vapor on the drift times of small ions at different temperatures was studied experimentally using an ion mobility spectrometer. The experiments were carried out for hydronium, ammonium, oxygen, chloride, bromide, and iodide ions. A theoretical model was developed, allowing us to calculate the effective mobility of ions for a given concentration of water vapor and temperature. The basic assumption adopted in this model was the linear dependence of the effective mobility coefficient on the mobility of ions with a certain degree of hydration. The weighting factors in this relationship are the abundances of individual types of ions. These parameters were determined by calculations based on the thermodynamics of the formation and disintegration of ionic clusters. From the known values of temperature, pressure, and humidity, the values of effective mobilities can be predicted quite accurately. The dependencies of reduced mobilities on the average degree of hydration were also determined. For these dependencies, the measurement points on the graphs are gathered along specific lines. This means that the average degree of hydration unambiguously determines the value of reduced mobility for a given type of ions.

## Introduction

Ion mobility spectrometry (IMS) is an analytical technique used primarily for the detection of hazardous materials. Differentiation of chemical compounds in IMS is based on the study of ion movement in gases under the influence of an electric field. These ions are formed from molecules of sample components or their fragments. The most commonly used detectors in the IMS technique are spectrometers with drift tubes (DT IMS), in which the time of ion movement through a specific drift distance is measured. An extensive description of the IMS technique can be found in the monograph,^1^ as well as in numerous scientific publications.^2−4^ Despite the fact that IMS detectors can be used alone as analytical devices, they can also be coupled to other analytical instruments e.g., gas and liquid chromatographs or mass spectrometers enhancing their analytical capabilities.^5−7^

The basic parameter characterizing the movement of ions in the gas is their mobility *K*, defined as the ratio of the ion drift velocity *v*_d_ to the electric field strength *E*. Mobility refers to the swarm of ions and determines the average velocity of their movement in the electric field. For ions of a certain shape and mass, the mobility can be estimated on the basis of the Mason–Schamp formula^8,9^
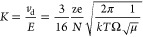
1where *ze* is the ion charge, *N* is the number density of the gas (number of molecules per unit volume), *k* is the Boltzmann constant, *T* is the temperature, *μ* represents the reduced mass, and Ω is the average collision cross section. The reduced mass is defined as the product of the masses of the ion *m* and the drift gas molecule *M* divided by the sum of these masses: *μ* = *m*·*M*/(*m* + *M*). In the case of IMS measurements, the estimation of mobility using eq 1 is possible for a limited group of ions (e.g., some dimer ions) or for unusual operating conditions of the spectrometer (e.g., at relatively high temperatures).

In real operating conditions of DT IMS, the ions change their composition during movement in the drift region. This occurs mainly due to the change in the degree of ions hydration. The number of water molecules attached to the core of a particular ion changes during the drift. For this reason, the reduced mass and the collision cross section, i.e., the values present in eq 1, cannot be explicitly determined. Changes in the degree of hydration are statistical in nature, and the number of attached water molecules results from the thermodynamics of the formation and dissociation processes of ion clusters.

The influence of ion–molecule reactions on the ion drift time was described by Tabrizchi,^10^ who considered two ion forms (positive monomer and dimer ions) in equilibrium with neutral molecules of a chemical compound forming these ions. In the drift time spectrum, a single peak is observed, and its position depends on the concentration of this compound. It was shown that a similar phenomenon can be observed for Cl^–^ ions and M·Cl^–^ clusters,^11^ in which M is an organic substance molecule solvating the chloride ion. It has been demonstrated that the measurements of changes in the effective drift time can be used to calculate the enthalpy of the clusterization process. A theoretical model of charge transport during which the ion composition changes was presented by Izadi.^12^ Quite a complex mathematical description, on which this model is based, leads to the conclusion that the inversed effective drift time is a weighted sum of the inversed individual drift times
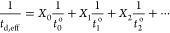
2The weighting factors in this dependence are mole fractions of individual ions *X*_*n*_ involved in the charge transport. Individual drift times *t*_*n*_^o^ are abstract quantities corresponding to ideal situation in which the charge is carried by one type of ions. In another paper, the Izadi model was used to analyze the mobility of hydrated hydronium and ammonium ions.^13^ Theoretical considerations in this work allowed the authors to introduce the concept of standard mobility, independent of the degree of ion hydration. The basis for the considerations leading to the definition of this quantity was the semiempirical dependence of the mobility on the mass of the ion. The influence of humidity on the drift times of negative ions was investigated by Mayer and Borsdorf.^14^ They determined the effect of humidity on peak-to-peak resolution for halide and oxygen ions.

The mobility values for various ions can also be calculated theoretically. The best known software that can be used for this purpose is MOBCAL.^15^ The use of this software requires knowledge of the ion structure, which is usually determined with Gaussian software. The theoretical mobility values of hydronium ions with different degrees of hydration, calculated using several methods, are summarized in the article by Gunzer.^16^

The presence of water in the gases flowing through IMS detectors is an important factor affecting the analytical performance of these instruments, and therefore extensive research is being carried out in this field. These works very often concern the influence of humidity on detection sensitivity. Usually, an increase in humidity causes a decrease in the ionization efficiency of analytes.^17,18^ In recent years, there has been a significant increase in interest in using differential ion mobility spectrometry (DMS) to study the properties of various ionic clusters, including ions with different degrees of hydration.^19,20^ The DMS technique seems to be a very effective tool for studying the kinetics and thermodynamics of clustering and declustering processes.^21^ Similar conditions as in DMS occur in drift tube detectors operating at reduced pressure. This measurement technique, called high kinetic energy IMS (HiKE-IMS), can be used to study the mobility of hydrated ions. The results obtained with this technique were analyzed by Erdogdu et al. using a theoretical model based on thermodynamic data and computer simulation.^22^

The aim of our work was to study the influence of humidity on the mobility of six types of small ions: the hydronium and ammonium ions were investigated in the positive mode, while the behavior of oxygen, chloride, bromide, and iodide ions was studied in the negative mode. A simple theoretical model of the transport phenomenon was developed, allowing us to calculate the effective mobility of ions for a given concentration of water vapor and temperature.

## Experimental Section

### DT IMS

The drift tube ion mobility spectrometer IMSD-B constructed at the Institute of Chemistry of the Military University of Technology was used in the research. Gas ionization in this device occurs due to the β radiation emitted from the ^63^Ni (300 MBq) radioactive source. The lengths of the reaction and drift detector regions are 5.7 and 6.1 cm, respectively. Ions are injected into the drift section using a Bradbury–Nielsen shutter grid. All measurements were carried out for a grid opening time of 0.15 ms, at an electric field strength in the drift section of 251 V·cm^–1^. The carrier and the drift gas flows were equal to 0.5 L·min^–1^. The DT IMS temperature was within the range of 318–378 K. In the tests carried out for hydronium and ammonium ions, the spectrometer worked in the positive mode. For the remaining types of ions, the measurements were conducted in the negative mode of operation. A description of the IMSD-B detector and a sketch of its reaction section can be found in our previous work.^23^

### Gas System

**Figure 1:**
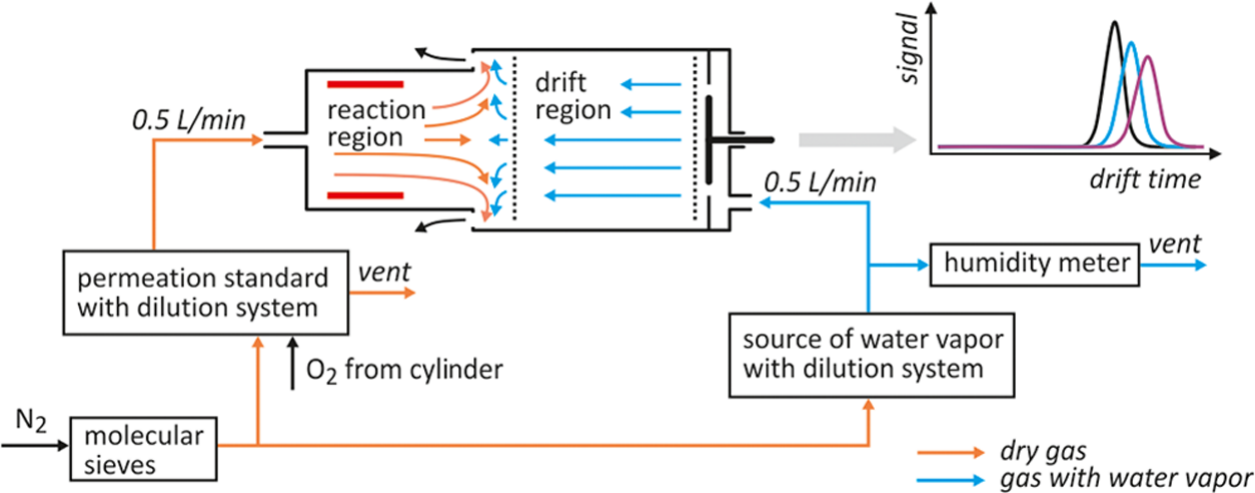
Measuring system diagram.

The diagram of the gas connection system used in the tests is shown in Figure 1. Carrier gas was introduced into the DT IMS reaction section. During tests performed for hydronium and ammonium ions, the carrier gas did not contain any intentionally introduced admixtures. Oxygen necessary for the formation of oxygen ions resulting from associative electron capture was supplied from a gas cylinder. Its concentration in the carrier gas was about 2 vol %. Halide ions were generated from halogen derivatives in the process of dissociative electron capture. Substances involved in this process (benzyl chloride, 1-bromohexane, and 1-iodobutane) were introduced to the carrier gas from permeation standards using a single-stage dilution system. In this system, Brooks mass flow controllers (SLA5850 and GFA40) and 0254 control modules were used. The concentration of halogen derivatives in the carrier gas was approx. 0.9 ppm for benzyl chloride, 7 ppm for 1-bromohexane, and 0.8 ppm for 1-iodobutane.

The system for water vapor introduction to the drift gas was constructed in a similar way. The source of water vapor was an open thermostated vessel containing distilled water. The water vapor concentration was controlled by a dilution system and determined using the source mass loss. In addition, a dev.*IQ* hygrometer (GE Sensing EMEA) was installed in the water vapor introduction system and used for control of water concentration. The construction of single-step dilution systems used to introduce admixtures to the carrier and drift gases was similar to those presented in the paper by Budzyńska et al.^24^

### Chemicals and Gases

Halogenated organic compounds, i.e., benzyl chloride (Sigma-Aldrich, purity 99%), 1-bromohexane (Sigma-Aldrich, purity 98%), and 1-iodobutane (Fluka, purity > 98%), were used in the research. Nitrogen, which was the carrier and drift gas, was produced using a NiGen HF-1 (CLAIND) nitrogen generator. Oxygen from a gas bottle (99.999%, MULTAX) was also used for the measurements. Molecular sieves with a pore diameter of 1.0 nm (Merck) placed in a 2 L container were used to purify and dry the gases.

## Results and Discussion

### Determination of Reduced Mobilities

**Figure 2:**
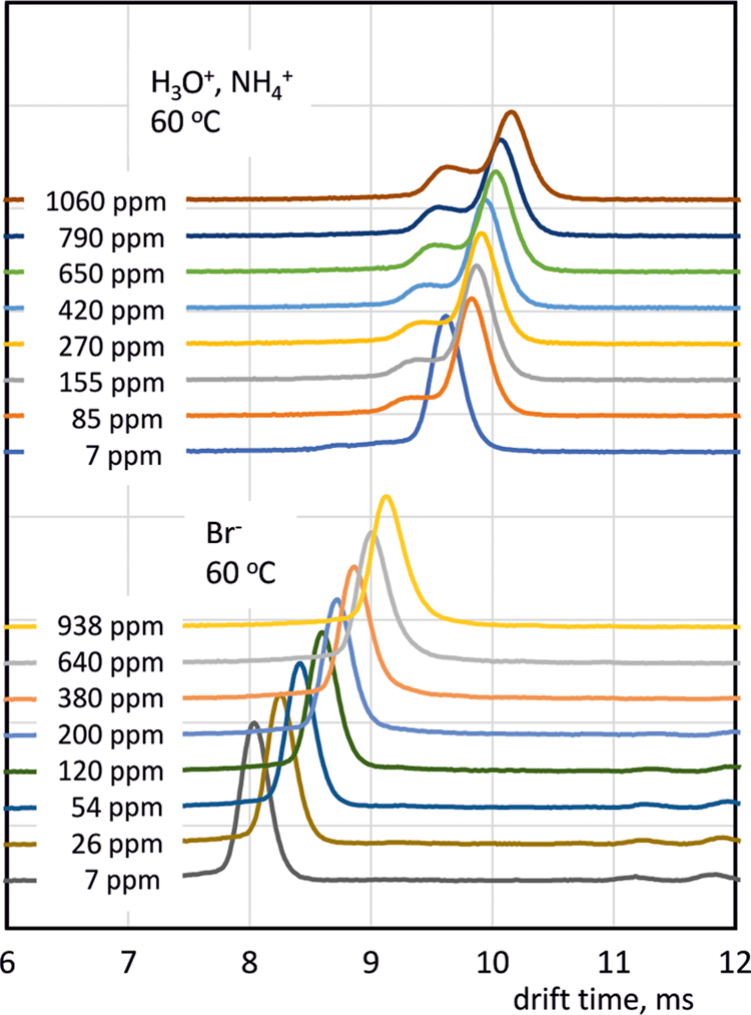
Example drift time spectra measured for hydronium, ammonium, and bromide ions at different water vapor concentrations.

In the measuring system (Figure 1), the drift time spectra for different temperatures and humidities for hydronium, ammonium, oxygen, chloride, bromide, and iodide ions were recorded. In total, about 240 measurements of drift time spectra were performed. The results of these measurements are collected in the Supporting Information (see Tables S1–S6). Exemplary spectra recorded for H_3_O^+^, NH_4_^+^, and Br^–^ are shown in Figure 2. For each spectrum, the ion drift time was determined, and then mobility *K* and reduced mobility *K*_0_ of ions were calculated
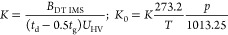
3where *t*_d_ is the drift time, *t*_g_ is the shutter grid opening time, *T* is the temperature, and *p* is the gas pressure in the detector in hPa. *U*_HV_ is the high voltage supplying the detector, i.e., the sum of the voltages on the reaction and drift sections. *B*_DT IMS_ is the detector constant, which depends on the length of the drift distance and the parameters of the voltage divider determining the potential distribution on the detector electrodes. The value of the constant *B*_DT IMS_, which is 76.3 cm^2^, was determined experimentally by calibration using a mobility standard. This standard was 2,6-di-tert-butylpyridine (DtBP), for which the value of reduced mobility determined on the basis of precise measurements by Hauck et al.^25^ is 1.477 cm^2^(Vs)^−1^. It should be noted that for many years the value of reduced mobility for DtBP was assumed^26^ to be 1.42 cm^2^(Vs)^−1^. In any case, it is important to realize that the results of the mobility calculations shown below are directly proportional to the assumed mobility of the standard.

**Figure 3:**
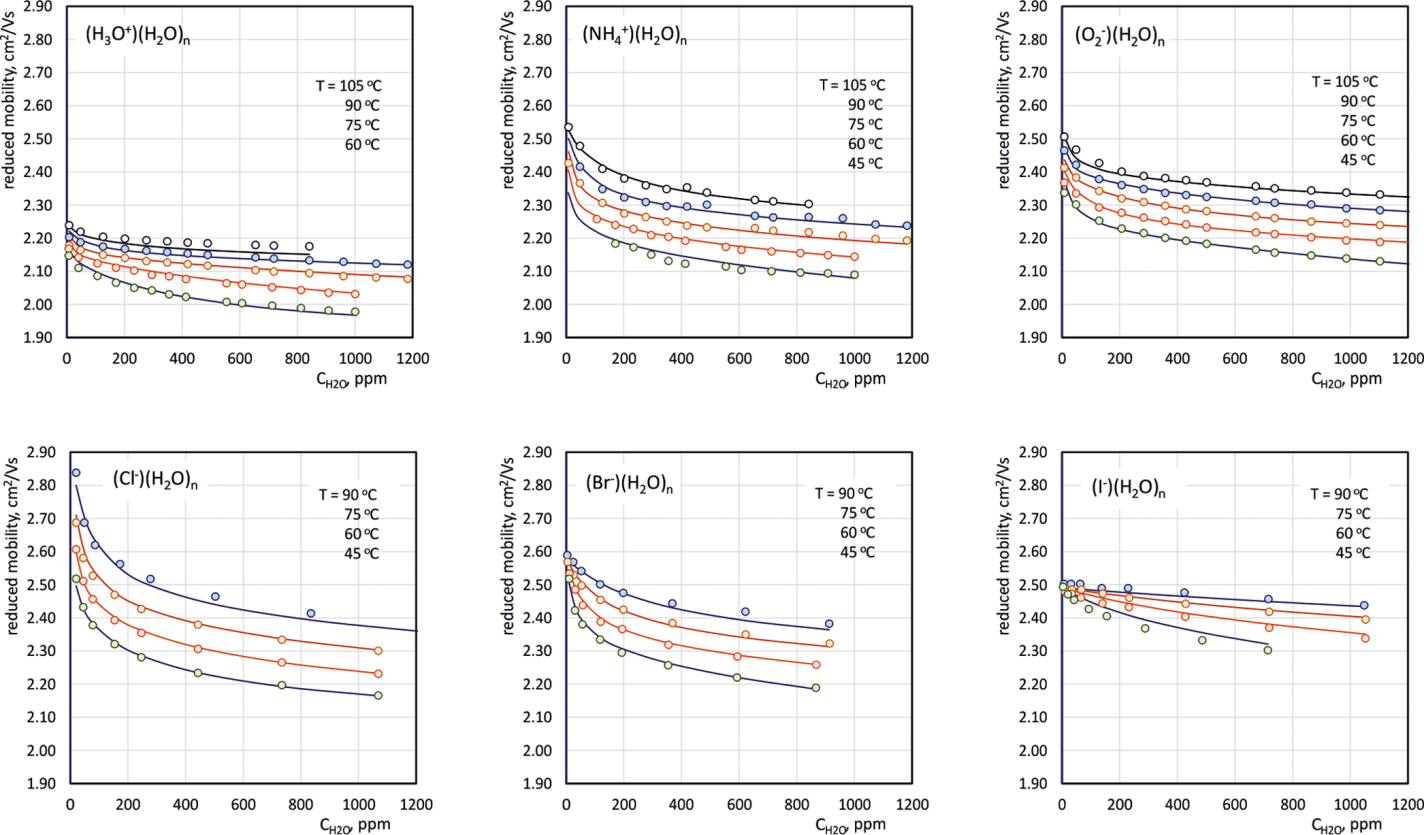
Dependencies between humidity and reduced mobilities for six kinds of small ions. Circles represent measurement data, and solid lines are values calculated based on the mobilities of ions with a particular number of water vapor. In all graphs, successive curves, starting from the bottom up, correspond to increasingly higher temperatures.

Values of reduced mobilities measured for hydronium, ammonium, oxygen, chloride, bromide, and iodide ions at various humidities and temperatures are shown (as circles) in the graphs in Figure 3.

### Calculation of Mobility for Ions with Different Degrees of Hydration

The main goal of the experimental studies and the analysis of their results was to determine the values of the reduced mobilities *K*_0*i*_ of ions with a certain hydration degree. The fundamental assumption adopted in the methodology of the analysis of results was that the effective reduced mobility is a linear combination of the ion mobilities with a certain degree of hydration, and the coefficients in this relationship are the abundances *A*_*i*_
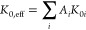
4This dependence results directly from eq 2 and is valid for reduced mobility as well as the mobility measured at a given temperature and pressure. Calculations of the effective mobility require knowledge of the ion abundance values with particular degrees of hydration for a given ion core *M* (e.g., H_3_O^+^, Cl^–^) at different temperatures and humidity. The values of the abundance are determined based on the equilibrium constants of the reaction of attaching successive water molecule to an ion with the degree of hydration *n*
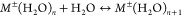
5The molar concentration of the product of this reaction can be determined by the equation
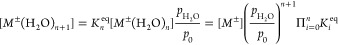
6where *K*_*n*_^eq^ is the reaction equilibrium constant, *p*_H_2_O_ is the partial pressure of water vapor, and *p*_0_ is the total gas pressure. The value of the abundance of ions with the hydration degree of *n* is equal to the molar concentration of a given ion type divided by the sum of the molar concentrations of ions with all possible degrees of hydration
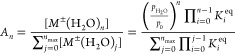
7The equilibrium constant can be determined from the change of free energy Δ*G*°, which is related to the enthalpy Δ*H*° and entropy Δ*S*° of the reaction
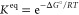
8
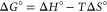
9where *T* is the temperature and *R* is the gas constant.

**Figure 4:**
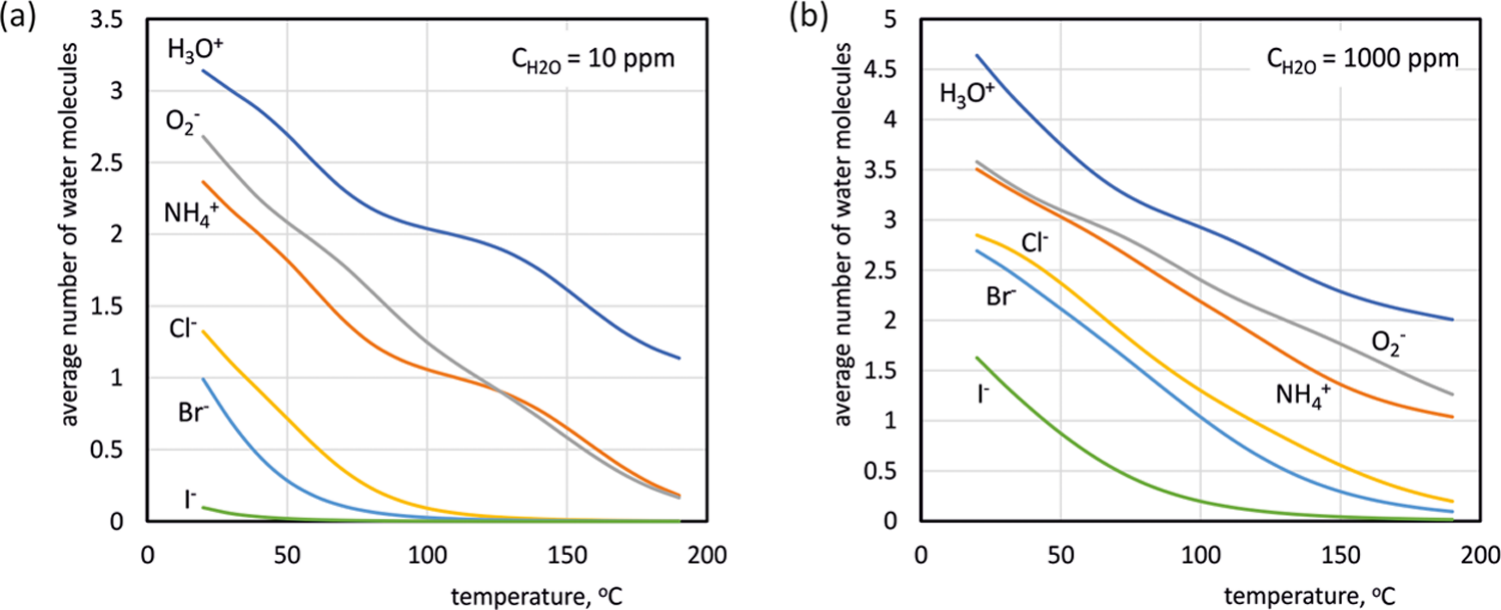
Average number of water molecules in clusters at water vapor content of 10 ppm (a) and 1000 ppm (b).

Thermodynamic data needed to determine the abundance are presented in Table 1. Calculations based on eqs 7–9 give very similar results to those published by Stone^27^ and Borsdorf.^28^Table 1 also includes the average values of the hydration degree *n*_avg_ obtained using the formula
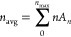
10These values are functions of temperature and water concentration in gases. Moreover, *n*_avg_ depends on the type of the ion core. Graphs of average hydration degree versus temperature, plotted for two different concentrations of water vapor, are shown in Figure 4. The concentration of 10 ppm (Figure 4a) corresponds to the typical content of water vapor introduced with the carrier and drift gas to stationary ion mobility spectrometers used in laboratories. In IMS detectors used for on-site analysis, the concentration of water vapor is significantly higher. For this reason, the graph in Figure 4b was drawn for a water vapor concentration of 1000 ppm. The abundances are functions of two quantities—temperature and humidity. It is possible to present this dependence in different ways (e.g., as 3D graphs). In the Supporting Information (see Figure S1), we included graphs in which the average hydration degree is shown as a function of humidity at temperatures of 45 and 90 °C.

**Table 1:** Thermodynamic Parameters Used in Calculations and Estimated Mobilities of Ion Clusters with Particular Number of Water Molecules

ion core	H_3_O^+^^a^	NH_4_^+^^b^	O_2_^–^b	Cl^–^b	Br^–^b	I^–^b
Enthalpy Change, −Δ*H*°, kJ/mol						
*n*: 0↔1	150.6	86.2	77.0	59.5	52.0	43.3
*n*: 1↔2	93.3	72.8	72.0	52.0	50.6	40.6
*n*: 2↔3	71.1	57.3	64.4	49.9	48.0	38.3
*n*: 3↔4	64.0	45.2	54.0			
*n*: 4↔5	54.4					
*n*: 5↔6	49.0					
Entropy Change, −Δ*S*°, J/(mol·K)						
*n*: 0↔1	139	103	84	83.0	73.8	71.0
*n*: 1↔2	121	120	105	87.4	85.6	79.3
*n*: 2↔3	118	104	118	94.7	99.0	86.7
*n*: 3↔4	136	96	124			
*n*: 4↔5	127					
*n*: 5↔6	124					
Average Number of Water Molecules in Cluster						
45 °C, 10 ppm	2.79	1.91	2.16	0.81	0.36	0.02
45 °C, 1000 ppm	3.88	3.10	3.16	2.48	2.22	0.98
	2.10	1.13	1.41	0.15	0.04	0.00
90 °C, 10 ppm	3.04	2.36	2.56	1.49	1.25	0.27
90 °C, 1000 ppm						
Reduced Mobilities of Clusters with *n* Molecules of Water, cm^2^/V·s						
*n* = 0				2.89	2.56	2.49
*n* = 1	2.65^c^	2.52	2.54	2.45	2.37	2.29
*n* = 2	2.22	2.30	2.39	2.28	2.28	2.15^c^
*n* = 3	2.14	2.14	2.22	2.06	1.90^c^	
*n* = 4	1.87^c^	1.76^c^	1.77^c^			

a Thermodynamic data from Stone.^27^

b Thermodynamic data from the Chemistry Webbook (http//webbook.nist.gov/).

c Abundances of these kinds of ions are small in measurement conditions.

The dependencies of the reduced mobilities on humidity are presented in the graphs in Figure 3. The measured values are marked on these graphs with points (circles). The solid lines correspond to the theoretical values obtained from eq 4. The mobility values for ions with a certain degree of hydration, required to perform calculations based on the equation above, were determined by minimizing the square of the difference between the measured and calculated effective mobility values
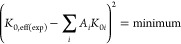
11Determination of the minimum was carried out by selecting the appropriate values of particular mobilities *K*_0*i*_ for all values of humidity and temperature at which measurements were carried out for a given type of ions. For hydronium, ammonium, and oxygen ions, fittings were carried out for *n* = 1–4; for chloride and bromide ions, for *n* = 0–3; and for iodide ions, for *n* = 0–2 (*n* is the number of water molecules in cluster). The fittings were made using the SOLVER function available in Microsoft EXCEL.^29^ The nonlinear GRG (generalized reduced gradient) method was used. A calculator for estimating the mobility of ions with different degrees of hydration is described in the Supporting Information (see Figures S2 and S3). This file also includes a description of the method for calculating the abundance of individual ions. The calculated values of the ions mobilities with a certain degree of hydration are presented in Table 1. The accuracy of these values determination depends on the abundances of the relevant ions in the entire range of temperatures and humidity in which the measurements for a given type of ions were made. It was assumed that if for a specific degree of hydration the abundance is less than 10%, the calculations may be inaccurate. Values of reduced mobilities for such ions presented in Table 1 have been marked with an appropriate index.

Analyzing the results presented in Figure 3, it can be concluded that the considered model of charge transport in the DT IMS drift section allows, quite precisely, for prediction of the effective mobilities for small ions that change their hydration degree during their movement. The results of the calculations are in most cases consistent with the data obtained experimentally. The reasons for the limited accuracy of the method may be related to both the measurement technique used and the basics of the theoretical model adopted to describe the phenomena. Experimental determination of reduced mobility is always associated with the occurrence of measurement uncertainties.^30^ These result from the construction of the detector and the measurement conditions, i.e., the values of the electric field, temperature, and pressure. In the case of the studies described in this paper, it was particularly difficult to precisely determine the content of water vapor in the drift gas at a low concentration level.

### Dependence of Effective Mobility on the Average Degree of Hydration

Theoretical problems associated with the presented method are mainly related to the thermodynamics of the formation and dissociation of ion clusters. It was assumed that these processes occur along the entire drift path in conditions of thermodynamic equilibrium. This assumption is correct if the time required to establish equilibrium is much shorter than the drift time. The fulfillment of this condition depends on the type of ions and the concentration of chemical individuals involved in the reactions (i.e., concentration of water vapor). Another problem is the quality of thermodynamic data, on the basis of which the abundances of individual ions are calculated. These data, given in various publications, differ from each other, which ultimately affects the determined values of the ion mobility with a certain degree of hydration.

**Figure 5:**
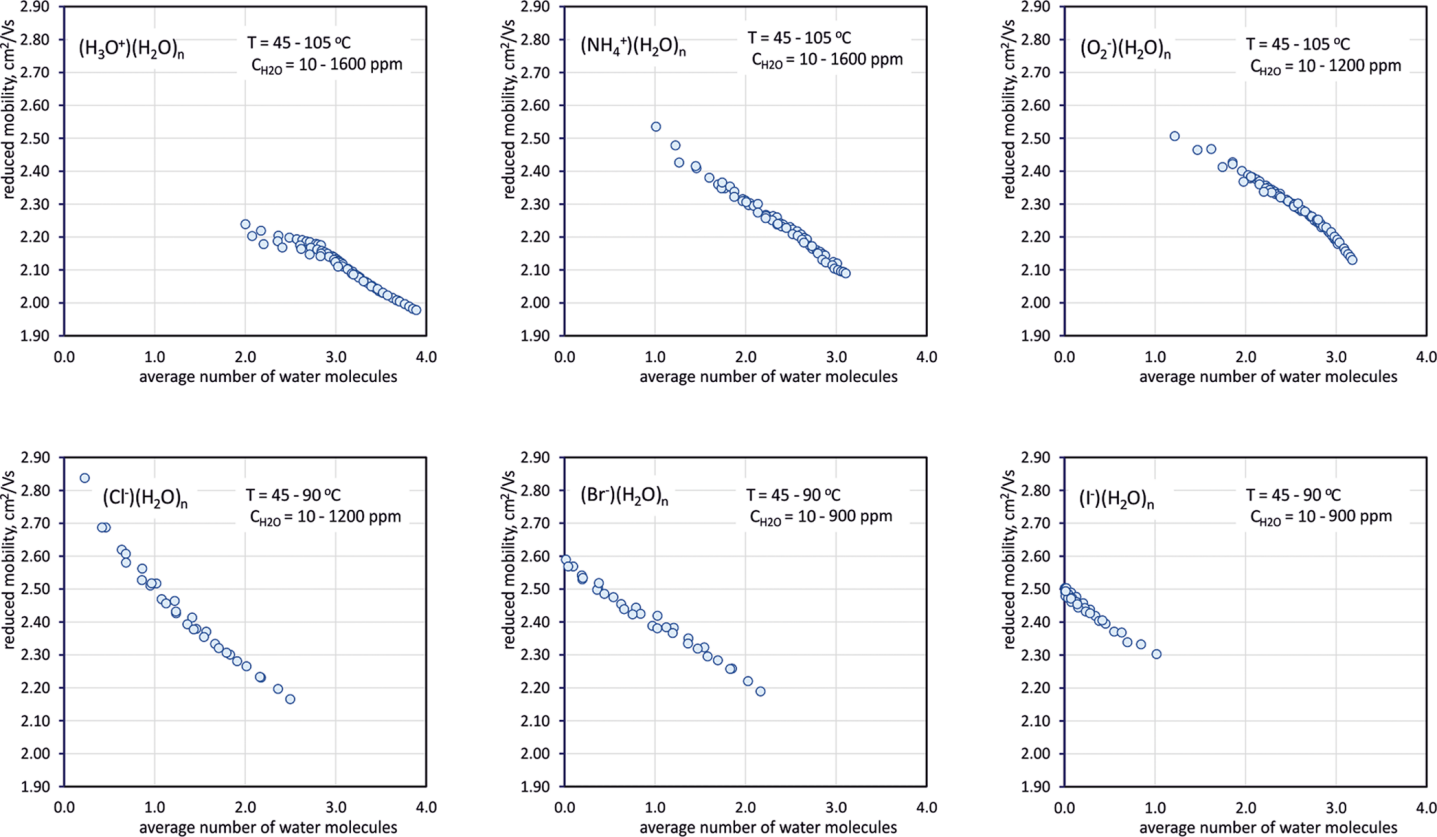
Dependencies of reduced mobilities on the average number of water molecules in ion clusters.

The graphs in Figure 3 contain reduced mobilities measured for different humidity and temperatures. Both of these factors contribute to the average degree of hydration. Therefore, the dependence of reduced mobilities on the hydration degree is interesting. Appropriate calculations were made for all measurement data, with the values of the average degrees of hydration determined using eq 10. The results of these calculations are shown in Figure 5.

A characteristic feature of obtained dependencies is that the measurement points on the graphs are gathered along specific lines. This means that the same effective mobilities can be observed at two different temperatures with appropriately “adjusted” humidity. It is only important that the average degree of hydration is the same. Therefore, it can be stated that the average degree of hydration determines the value of reduced mobility for a given type of ions. Another interesting observation regarding the results shown in Figure 5 is that the slope of the relationship is almost the same for different types of ions (about 0.2 cm^2^V^–1^s^–1^ per unit change in the degree of hydration). The graphs presented in Figure 5 show a greater dispersion of measurement points for lower degrees of hydration. The reason for this phenomenon may be the not very high precision of the measurement of water concentration for low humidity.

## Conclusions

The presence of water in gases flowing through IMS detectors has a significant impact on the qualitative and quantitative aspects of the use of these instruments in analytics. For many types of ions, peaks shifting in the drift time spectrum is observed due to the changing concentration of water vapor. The dependencies presented in Figure 3 show that the changes in mobility for various types of ions are different. However, the greatest relative changes are always observed for relatively low values of water concentration (5–100 ppm). This humidity range corresponds to the conditions found in detectors used in laboratory studies. The theoretical analysis of the dependence between reduced mobility and water concentration was based on the assumption that the effective mobility coefficient is a linear function of the mobility of individual ion forms, which take part in charge transport. Moreover, the coefficients included in this relationship are the abundances of individual types of ions (eq 4). Using thermodynamic data, the abundances and reduced mobilities of individual types of ions were determined. The theoretical values of effective mobility calculated on this basis are close to the data obtained experimentally. This means that with known values of temperature, pressure, and humidity, the values of effective mobilities can be predicted quite accurately. The reverse approach is also possible, i.e., moisture estimation based on ion mobility. This idea has already been tested by Hauck et al.,^31^ who used the change in the mobility of protonated molecules to determine moisture. The information obtained in this way may be important for quantitative research because very often the presence of water vapor significantly affects the detection sensitivity. Knowledge of the humidity value will enable the introduction of appropriate corrections to the algorithms that allow concentration determination based on the analytical signal generated in the IMS detector.

## Supporting Information Available

Measured values of effective mobilities; average number of water molecules in clusters at given temperatures; and calculators for estimation of ion abundances and mobilities (PDF)
