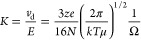# Correction to “Effect of Humidity on the Mobilities of Small Ions in Ion Mobility Spectrometry”

**DOI:** 10.1021/acs.analchem.3c02667

**Published:** 2023-07-01

**Authors:** Izabela Wolańska, Krzysztof Piwowarski, Edyta Budzyńska, Jarosław Puton

We would like to correct the form in which eq 1 appears in our original paper. During the editing process, a serious typographical error has been introduced into the formula. Some parameters in the equation were incorrectly entered under the square root symbol. The correct form of eq 1 is as follows: