# Supplementary material for: Effect of Humidity on the Mobilities of Small Ions in Ion Mobility Spectrometry
Source: Anal Chem. 2023 May 23;95(22):8505–11. doi: 10.1021/acs.analchem.3c00435 (PMC10248996; doi:10.1021/acs.analchem.3c00435)
Supplement: Supplementary file 1 — ac3c00435_si_001.pdf [file ac3c00435_si_001.pdf]

Supporting Information

The Effect of Humidity on the Mobilities of Small Ions in Ion Mobility Spectrometry

Izabela Wolańska, Krzysztof Piwowarski, Edyta Budzyńska, Jarosław Puton*

Faculty of Advanced Technologies and Chemistry, Military University of Technology, ul. gen. Sylwestra Kaliskiego 2, 00-908 Warsaw, Poland

* Corresponding author: Email: jaroslaw.puton@wat.edu.pl

Table of contents

Table S1: Effective mobilities measured for $(H_3O^+)(H_2O)_n$ page S-2

Table S2: Effective mobilities measured for $(NH_4^+)(H_2O)_n$ page S-2

Table S3: Effective mobilities measured for $(O_2^-)(H_2O)_n$ page S-3

Table S4: Effective mobilities measured for $(Cl^-)(H_2O)_n$ page S-3

Table S5: Effective mobilities measured for $(Br^-)(H_2O)_n$ page S-4

Table S6: Effective mobilities measured for $(I^-)(H_2O)_n$ page S-4

Figure S1: Average number of water molecules in clusters at given temperatures page S-5

Figure S2: EXCEL worksheet used for determination of the abundance of ions with specific degrees of hydration. page S-7

Figure S3: EXCEL worksheet used for estimation of the reduced mobility that can be attributed to different types of ions page S-8

Tables S1-S6: Measured values of effective mobilities

Table S1: Effective mobilities measured for $(H_3O^+)(H_2O)_n$

$(H_3O^+)(H_2O)_n$									
45°C		60°C		75°C		90°C		105°C	
C_{H2O}	$K_{0,exp}$	C_{H2O}	$K_{0,exp}$	C_{H2O}	$K_{0,exp}$	C_{H2O}	$K_{0,exp}$	C_{H2O}	$K_{0,exp}$
7	2,148	7	2,168	8	2,179	8	2,204	8	2,239
41	2,110	41	2,142	48	2,164	48	2,188	48	2,219
107	2,086	107	2,125	126	2,150	126	2,175	126	2,204
171	2,065	171	2,111	202	2,140	202	2,167	202	2,199
234	2,050	234	2,103	276	2,131	276	2,162	276	2,193
295	2,043	295	2,089	348	2,127	348	2,158	348	2,190
355	2,031	355	2,085	419	2,122	419	2,154	419	2,187
413	2,023	413	2,077	488	2,117	488	2,150	488	2,185
554	2,008	554	2,064	654	2,104	654	2,142	654	2,180
608	2,004	608	2,060	718	2,100	718	2,138	718	2,178
712	1,997	712	2,052	842	2,095	842	2,133	842	2,176
812	1,990	812	2,044	960	2,086	960	2,129		
908	1,982	908	2,035	1073	2,081	1073	2,124		
1000	1,978	1000	2,031	1182	2,077	1182	2,121		
		1398	2,016						
		1629	2,007						

Table S2: Effective mobilities measured for $(NH_4^+)(H_2O)_n$

$(NH_4^+)(H_2O)_n$									
45°C		60°C		75°C		90°C		105°C	
C_{H2O}	$K_{0,exp}$	C_{H2O}	$K_{0,exp}$	C_{H2O}	$K_{0,exp}$	C_{H2O}	$K_{0,exp}$	C_{H2O}	$K_{0,exp}$
7		7		8	2,426	8		8	2,536
41		41		48	2,366	48	2,416	48	2,478
107		107	2,258	126	2,307	126	2,348	126	2,410
171	2,184	171	2,240	202	2,274	202	2,322	202	2,381
234	2,173	234	2,229	276	2,264	276	2,310	276	2,360
295	2,151	295	2,209	348	2,251	348	2,297	348	2,348
355	2,132	355	2,204	419	2,238	419	2,295	419	2,354
413	2,123	413	2,192	488	2,233	488	2,302	488	2,338
554	2,114	554	2,174	654	2,231	654	2,268	654	2,315
608	2,104	608	2,164	718	2,222	718	2,263	718	2,311
712	2,101	712	2,160	842	2,218	842	2,264	842	2,304
812	2,097	812	2,156	960	2,208	960	2,260		
908	2,095	908	2,149	1073	2,199	1073	2,242		
1000	2,090	1000	2,144	1182	2,193	1182	2,238		
		1398	2,125						
		1629	2,120						

Table S3: Effective mobilities measured for $(O_2^-)(H_2O)_n$

$(O_2^-)(H_2O)_n$									
45°C		60°C		75°C		90°C		105°C	
C_{H2O}	$K_{0,exp}$	C_{H2O}	$K_{0,exp}$	C_{H2O}	$K_{0,exp}$	C_{H2O}	$K_{0,exp}$	C_{H2O}	$K_{0,exp}$
12	2,247	12	2,277	12	2,320	12	2,370	12	2,410
53	2,213	53	2,245	53	2,291	53	2,329	53	2,373
133	2,166	133	2,205	133	2,253	133	2,287	133	2,334
211	2,143	211	2,190	211	2,231	211	2,269	211	2,309
287	2,130	287	2,175	287	2,220	287	2,258	287	2,296
362	2,117	362	2,165	362	2,210	362	2,247	362	2,290
435	2,108	435	2,156	435	2,199	435	2,241	435	2,284
506	2,099	506	2,146	506	2,194	506	2,235	506	2,278
676	2,082	676	2,132	676	2,179	676	2,224	676	2,266
742	2,074	742	2,128	742	2,174	742	2,219	742	2,260
869	2,065	869	2,118	869	2,164	869	2,213	869	2,254
990	2,057	990	2,109	990	2,159	990	2,202	990	2,248
1107	2,049	1107	2,105	1107	2,154	1107	2,197	1107	2,242
1218	2,040	1218	2,095	1218	2,144	1218	2,191	1218	2,242

Table S4: Effective mobilities measured for $(Cl^-)(H_2O)_n$

$(Cl^-)(H_2O)_n$							
45°C		60°C		75°C		90°C	
C_{H2O}	$K_{0,exp}$	C_{H2O}	$K_{0,exp}$	C_{H2O}	$K_{0,exp}$	C_{H2O}	$K_{0,exp}$
17	2,518	17	2,607	17	2,687	17	2,838
42	2,432	42	2,512	42	2,581	46	2,687
75	2,377	75	2,456	75	2,527	83	2,620
151	2,321	151	2,393	151	2,470	170	2,563
243	2,282	243	2,355	243	2,427	275	2,518
440	2,234	440	2,307	440	2,380	500	2,465
731	2,196	731	2,266	731	2,334	832	2,414
1065	2,165	1065	2,231	1065	2,302	1214	2,371

Table S5: Effective mobilities measured for $(Br^-)(H_2O)_n$

$(Br^-)(H_2O)_n$							
45°C		60°C		75°C		90°C	
C_{H2O}	$K_{0,exp}$	C_{H2O}	$K_{0,exp}$	C_{H2O}	$K_{0,exp}$	C_{H2O}	$K_{0,exp}$
11	2,518	12	2,533	5	2,569	4	2,590
30	2,423	32	2,486	26	2,529	25	2,569
56	2,381	58	2,439	54	2,498	52	2,542
118	2,335	120	2,388	119	2,454	118	2,501
193	2,295	194	2,366	199	2,424	198	2,475
354	2,257	355	2,319	369	2,384	368	2,444
593	2,220	594	2,284	623	2,350	622	2,419
866	2,189	868	2,259	914	2,322	912	2,383

Table S6: Effective mobilities measured for $(I^-)(H_2O)_n$

$(I^-)(H_2O)_n$							
45°C		60°C		75°C		90°C	
C_{H2O}	$K_{0,exp}$	C_{H2O}	$K_{0,exp}$	C_{H2O}	$K_{0,exp}$	C_{H2O}	$K_{0,exp}$
6	2,494	10	2,479	11	2,492	7	2,502
30	2,471	34	2,473	35	2,486	31	2,502
63	2,454	66	2,462	67	2,486	63	2,502
139	2,426	141	2,444	141	2,473	138	2,490
233	2,406	232	2,433	233	2,461	229	2,490
433	2,368	428	2,403	429	2,443	425	2,476
730	2,333	719	2,371	719	2,419	716	2,458
1071	2,303	1052	2,339	1053	2,395	1049	2,438

Figure S1: Average number of water molecules in clusters at given temperatures

The article contains graphs showing the dependence of the average value of the degree of hydration on temperature at fixed humidity values (see Fig. 4). Changes in the average value of the degree of hydration can also be presented as a function of humidity. Graphs of such relationships for temperatures of 45 and 90 °C are shown in Figure S1. As in the previous case, the calculations were carried out on the basis of formulas (5) - (8).

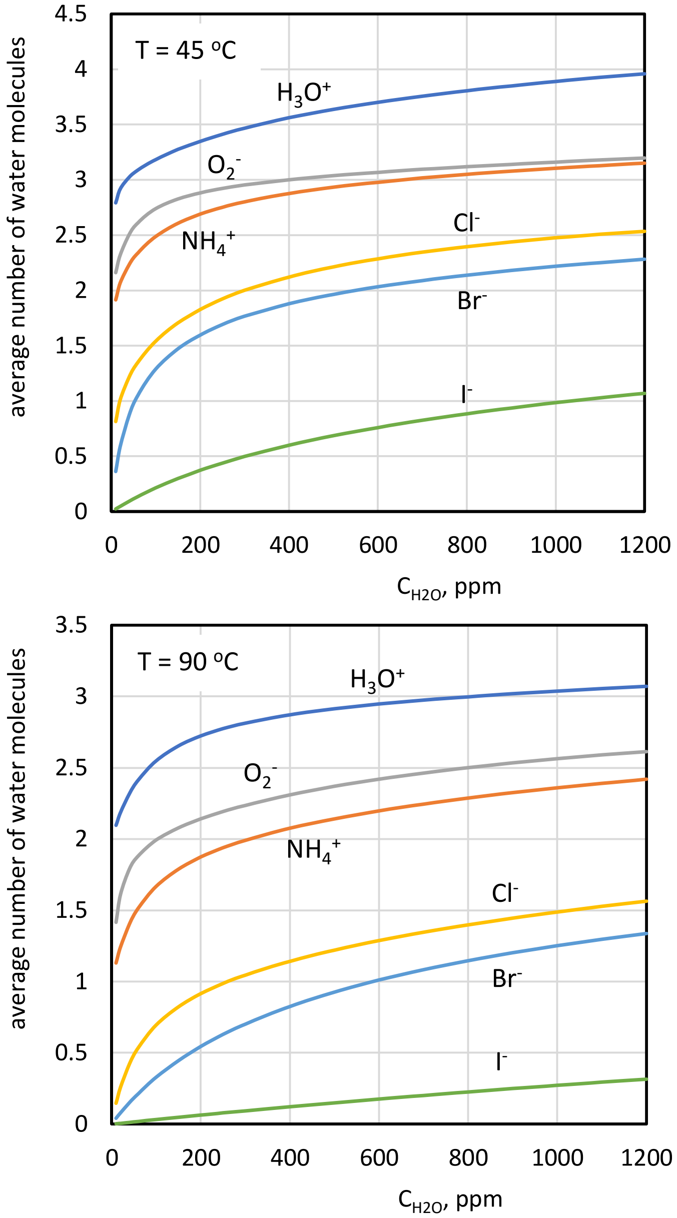

Figure S1: Average number of water molecules in clusters as a function of water vapor content at temperatures of 45 and 90 °C.

Figure S2, S3: Calculators for estimation of ion abundances and mobilities

The most important calculations that were carried out as part of our work concern the determination of the abundance of ions with specific degrees of hydration and the estimation of the mobility that can be attributed to different types of ions. Both types of calculations were performed in EXCEL spreadsheets. Below is a brief description of these calculations.

<u>Ion abundances with a certain degree of hydration.</u>

The view of the EXCEL sheet is shown in Figure S2. The sheet contains 3 tables. The first one from the left contains literature thermodynamic data. The middle table is the "working area" in which subsequent concentration values are calculated based on the equilibrium constants (according to the formula (5)). In the table on the right, there are normalized (see formula (6)) concentration values, i.e., abundances. The input values for the calculations are: the concentration of water vapor and the values of thermodynamic parameters.

<u>Reduced mobilities of ions with a certain degree of hydration.</u>

The EXCEL calculator for determining the mobility of individual ion species is shown in Figure S3. The input data for the calculations are:

i. experimentally determined values of reduced effective mobilities for specific humidity,

ii. table of ions with different degrees of hydration corresponding to these humidity levels.

The theoretical mobility value is calculated according to the formula (3), in which the mobilities of individual ionic forms are variable parameters of the optimization procedure carried out according to the formula (11) (blue numbers in Figure S3). The "energy function" (red number) is the sum of the squared differences between the experimental and theoretical mobility values for all humidity and temperature at which the measurements were made.

Sample spreadsheets (.xlsx files) shown in Figure S2 and S3 are available from the corresponding author.

(I) Determination of ion abundances

C_{H2O}	10	ppm	

	0↔1		1↔2		2↔3
ΔH_r	51.99	ΔHr	50.64	ΔHr	48
ΔS_r	73.8	ΔSr	85.55	ΔSr	99.05

Thermodynamic data used in calculations for Br^- with particular number of water molecules (n)*

n	0↔1	1↔2	2↔3
ΔH_r, kJ/mol	53.14	48.5	47.7
	49	51.04	28.9
	52.72	51.5	48.1
	53.1	51.5	48.1
**$\Delta H_{r,\ average}$**	**51.99**	**50.635**	**43.2**
ΔS_r, J/(mol·K)	61.5	75.3	94.1
	77	95.8	104
	82.8		
**$\Delta S_{r,\ average}$**	**73.76667**	**85.55**	**99.05**
ΔG_r, kJ/mol	30.5	26	20
	29	23	17
	29	22.6	17.2
		24	18
**$\Delta G_{r,\ average}$**	**29.5**	**23.9**	**18.05**

*http//webbook.nist.gov

ΔG_r = -RT lnK	
**R,** J/(mol·K)	8.314

T, °C	conc. [n=0]	K	conc. [n=1]	K	conc. [n=2]	K	conc. [n=3]	conc. total
20	1	255555.553	2.55555553	35742.9232	0.91343025	2385.91204	0.02179364	4.49077943
30	1	126471.464	1.26471464	18014.818	0.22783604	1246.25484	0.00283942	2.4953901
40	1	65464.7645	0.65464764	9485.73484	[illegible]	[illegible]	[illegible]	[illegible]
50	1	35295.5343	0.35295534	5196.96314	[illegible]	[illegible]	[illegible]	[illegible]
60	1	19748.5687	0.19748569	2951.98297	[illegible]	[illegible]	[illegible]	[illegible]
70	1	11430.0343	0.11430034	1732.9758	[illegible]	[illegible]	[illegible]	[illegible]
80	1	6823.50068	0.06823501	1048.50319	[illegible]	[illegible]	[illegible]	[illegible]
90	1	4190.86784	0.04190868	652.174299	[illegible]	[illegible]	[illegible]	[illegible]
100	1	2642.08017	0.0264208	416.110148	[illegible]	[illegible]	[illegible]	[illegible]
110	1	1706.25962	0.0170626	271.793143	[illegible]	[illegible]	[illegible]	[illegible]
120	1	1126.68752	0.01126688	181.41664	[illegible]	[illegible]	[illegible]	[illegible]
130	1	759.455647	0.00759456	123.544707	[illegible]	[illegible]	[illegible]	[illegible]
140	1	521.786431	0.00521786	85.7131201	[illegible]	[illegible]	[illegible]	[illegible]
150	1	364.910883	0.00364911	60.5026082	[illegible]	[illegible]	[illegible]	[illegible]
160	1	259.448417	0.00259448	43.3995152	[illegible]	[illegible]	[illegible]	[illegible]
170	1	187.326864	0.00187327	31.6014424	[illegible]	[illegible]	[illegible]	[illegible]
180	1	137.211786	0.00137212	23.3350729	[illegible]	[illegible]	[illegible]	[illegible]
190	1	101.864017	0.00101864	17.4581351	[illegible]	[illegible]	[illegible]	[illegible]
200	1	76.5804801	0.0007658	13.222466	[illegible]	[illegible]	[illegible]	[illegible]
210	1	58.2564201	0.00058256	10.1303004	5.9016E-08	1.035216	6.1094E-13	1.00058262
220	1	44.8111385	0.00044811	7.84555367	3.5157E-08	0.81249166	2.8565E-13	1.00044815

Values you can change are highlighted in yellow.

abundances of $Br^-(H_2O)_n$

T, °C	n=0	n=1	n=2	n=3
20	0.22267849	0.56906726	0.20340127	0.00485298
30	0.40073895	0.50682041	0.09130278	0.00113787
40	[illegible]	[illegible]	0.03616313	0.00024538
50	[illegible]	[illegible]	0.01337566	5.1308E-05
60	[illegible]	[illegible]	0.00484468	1.0872E-05
70	[illegible]	[illegible]	0.00177446	2.4035E-06
80	[illegible]	[illegible]	0.0006693	5.6306E-07
90	[illegible]	[illegible]	0.00026226	1.4067E-07
100	[illegible]	[illegible]	0.0001071	3.7521E-08
110	[illegible]	[illegible]	4.5595E-05	1.0668E-08
120	[illegible]	[illegible]	2.0212E-05	3.2238E-09
130	[illegible]	[illegible]	9.3119E-06	1.0319E-09
140	[illegible]	[illegible]	4.4492E-06	3.4865E-10
150	[illegible]	[illegible]	2.1998E-06	1.2391E-10
160	[illegible]	[illegible]	1.1231E-06	4.617E-11
170	[illegible]	[illegible]	5.9087E-07	1.7983E-11
180	[illegible]	[illegible]	3.1975E-07	7.3001E-12
190	[illegible]	[illegible]	1.7765E-07	3.0808E-12
200	[illegible]	[illegible]	1.0118E-07	1.3483E-12
210	0.99941772	0.00058222	5.8981E-08	6.1058E-13
220	0.99955205	0.00044791	3.5141E-08	2.8552E-13

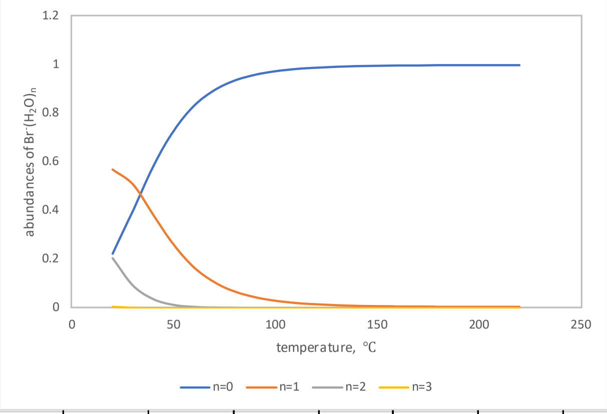

Figure S2: EXCEL worksheet used for determination of the abundance of ions with specific degrees of hydration.

(II) Calculator for estimation of reduced mobility for ions with given number of water molecules

Input: experimental Ko, abundances | Output: reduced mobilities | Solver: optimized value = I5, changed parameters: C5 - F5

T = 90°C

K_o(90°C)	2.488802966	2.2662293	2.2942265	1.79
K_o	2.4805	2.2817	2.1898	1.8275

sum of squares (T= 90-45°C)	
$\Sigma(K_{0,exp}-K_{0,theo})^2$	0.002985904

T = 75°C

K_o(75°C)	2.473102599	2.2778453	2.2206677	1.79
K_o	2.4805	2.2817	2.1898	1.8275

T = 90°C:

		abundances of $Br^-(H_2O)_n$					
C_{H2O}, ppm	$K_{o,exp}$, cm²/Vs	0	1	2	3	$K_{o,theo}$, cm²/Vs	$(K_{0,exp}-K_{0,theo})^2$
3.8	2.490003884	0.9842861	0.015675	3.885E-05	7.918E-09	2.477395165	0.00015898
24.669862	2.469882641	0.9049329	0.0935598	0.0015053	1.992E-06	2.461478543	7.06289E-05
52.468835	2.443554778	0.8147033	0.179149	0.0061304	1.725E-05	2.443102689	2.04385E-07
117.71284	2.405098834	0.653011	0.3221076	0.0247253	0.0001561	2.409175357	1.6618E-05
197.7158	2.38012701	0.5163971	0.4278528	0.0551651	0.000585	2.379015269	1.23597E-06
368.14031	2.349632109	0.3424295	0.5282517	0.1268149	0.0025039	2.336963503	0.000160494
621.7012	2.325793102	0.2128976	0.5546968	0.2249056	0.0075	2.299921635	0.000669333
912.40838	2.290927996	0.1386886	0.5303102	0.3155578	0.0154434	2.273226616	0.000313339
						$\Sigma(K_{0,exp}-K_{0,theo})^2$	0.001390832

T = 75°C:

		abundances of $Br^-(H_2O)_n$					
C_{H2O}, ppm	$K_{o,exp}$, cm²/Vs	0	1	2	3	$K_{o,theo}$, cm²/Vs	$(K_{0,exp}-K_{0,theo})^2$
4.9	2.469837764	0.9584075	0.0413205	0.0002719	1.418E-07	2.472227202	5.70941E-06
25.985866	2.432154289	0.808553	0.1849697	0.0064593	1.787E-05	2.441849087	9.39891E-05
53.85771	2.401618767	0.6629644	0.3141777	0.0227277	0.0001302	2.41135006	9.46981E-05
119.1304	2.360134915	0.4509213	0.4725321	0.0755888	0.0009577	2.363948261	1.45416E-05
198.7394	2.331370321	0.3097085	0.5422817	0.1449414	0.0030684	2.328534378	8.04257E-06
369.07915	2.292258057	0.1689475	0.5485256	0.2718552	0.0106718	2.285426876	4.6665E-05
622.8012	2.259762963	0.0879323	0.4820092	0.4033274	0.0267311	2.249941538	9.64604E-05
914.0348	2.233379274	0.0503159	0.4046416	0.4967422	0.0483002	2.224081245	8.64533E-05
						$\Sigma(K_{0,exp}-K_{0,theo})^2$	0.00044656

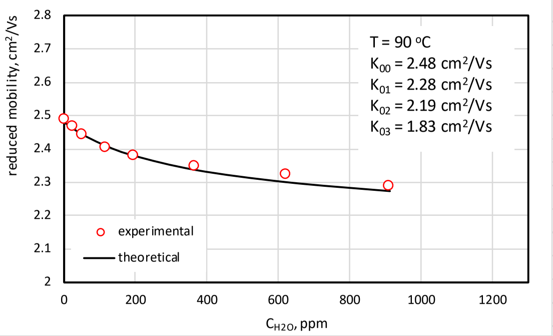

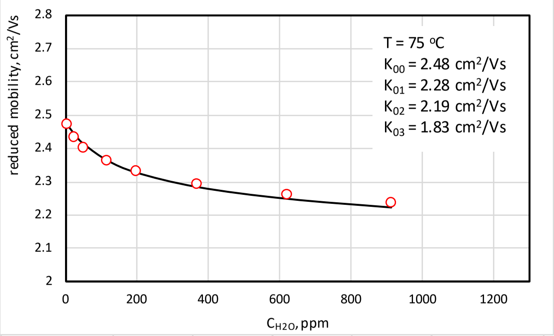

Figure S3: EXCEL worksheet used for estimation of the reduced mobility that can be attributed to different types of ions.
